# Olfactory binding proteins: a review across the Insecta

**DOI:** 10.1186/s12983-025-00584-0

**Published:** 2025-10-03

**Authors:** Ruisheng Yang, Jiani Zhou, Jiaxin Hao, Tiantao Zhang, Yiren Jiang, Wei Liu, Yong Wang

**Affiliations:** 1 College of Bioscience and Biotechnology, Shenyang Agricultural University, No. 120 Dongling Road, Shenhe District, Shenyang, 110866 Liaoning Province China; 2 Insect Resource Innovation Center for Professional Technology of Liaoning Province, No. 120 Dongling Road, Shenhe District, Shenyang, 110866 Liaoning Province China; 3 State Key Laboratory for the Biology of the Plant Diseases and Insect Pests, Institute of Plant Protection, Chinese Academy of Agricultural Sciences, Beijing, 100193 China

**Keywords:** Insect olfaction, Olfactory binding proteins, OBPs, CSPs, NPC2

## Abstract

Olfactory binding proteins are essential components of the highly sensitive olfactory system in insects. They play crucial roles in detecting, binding, and transporting environmental odorants and pheromones to olfactory receptors. Although a large number of olfactory binding proteins have been identified in insects to date, research in this field continues to advance rapidly. This review summarizes recent progresses in understanding their structures, functions, mechanisms of action, and potential applications. Structurally, these proteins typically form simple, stable, spherical conformations composed of α-helices and/or β-sheets, which support environmental adaptability and diverse physiological functions. Two main hypotheses have been proposed to explain their mechanisms of action: pH-dependent regulation and ligand-induced conformational changes. In terms of practical applications, olfactory binding proteins have shown great promise in biological pest control, the breeding of economically important insects, and the development of biosensors, making them attractive targets for future research and innovation.

## Introduction

Insects are the most diverse and widely distributed group of animals on Earth, playing a crucial role in natural ecosystems. More than one million insect species have been identified, accounting for over 80% of the phylum Arthropoda, outnumbering all other animal groups combined [1]. Their evolutionary success in complex environments is closely linked to their highly sophisticated and sensitive olfactory recognition system, which consists of both peripheral and central components [2–6]. The sensory system helps insects detect and process odorants, enabling key behaviors like mating, host finding, predator avoiding, and communicating [7–11].

**Fig. 1:**
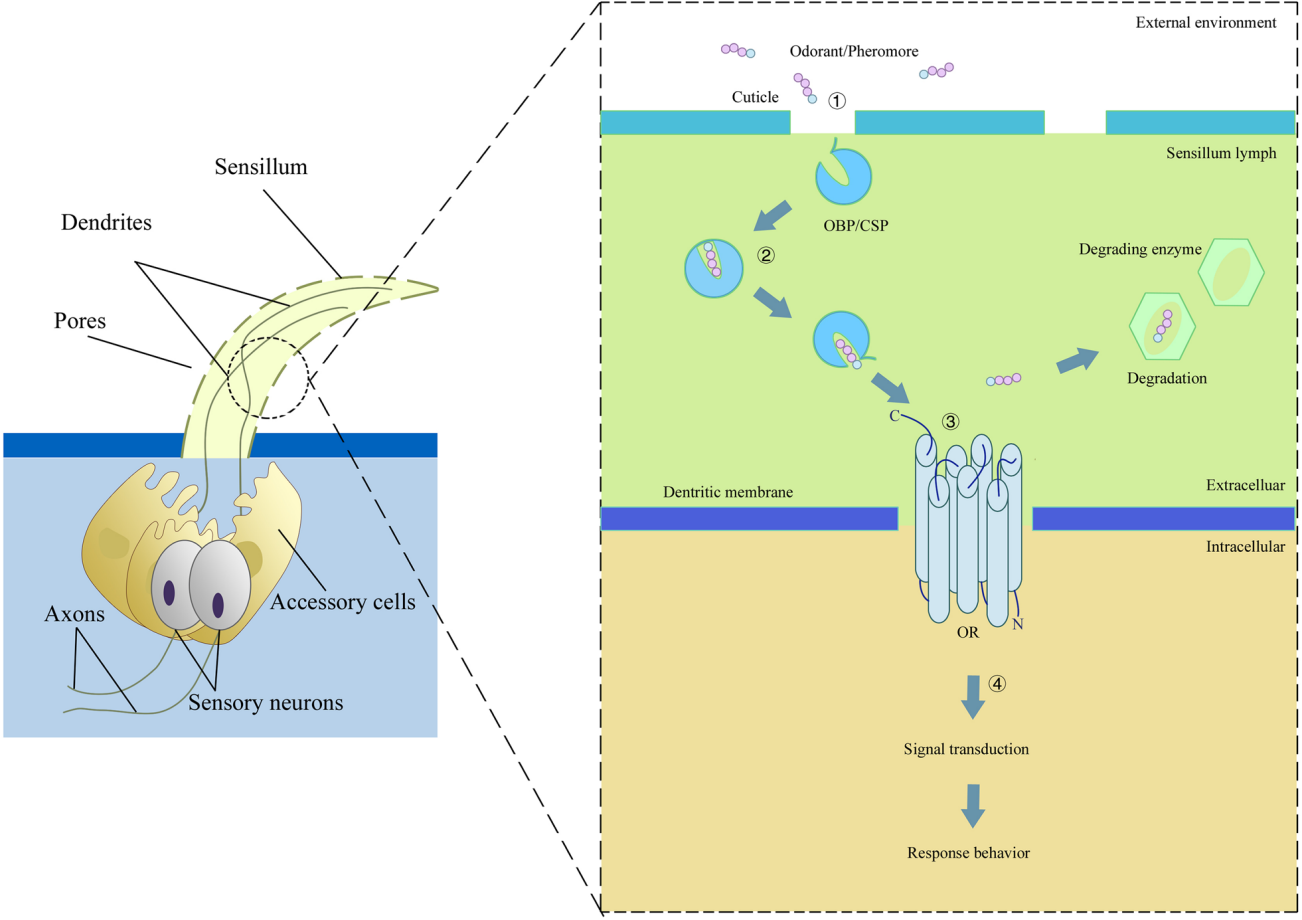
Schematic diagram of olfactory reception in insects. ① Odorant molecules in external environment diffuse into specialized sensilla through pore tubules. ② The odorants are recognized and bound by olfactory binding proteins (e.g. OBPs/CSPs), transported through the sensillum lymph to ORs. ③ Upon binding to the ORs, the signal transduction occurs within the olfactory neurons, and the remaining odorants are subsequently degraded by ODEs. ④ The electrical signal is transmitted to higher-order olfactory centers, resulting in corresponding response behavior

The link between behavioral responses and insect olfactory recognition involves a sequence of coordinated steps (Fig. 1). First, odorant or pheromone molecules in the environment diffuse into specialized sensilla through pores in the sensillar cuticle. Next, these molecules interact with olfactory binding proteins and are transported through the hemolymph within the sensilla to odorant receptors (ORs) located on the dendritic membranes of peripheral neurons [12]. Upon binding to the ORs, the chemical signal is converted into an electrical signal within the olfactory neurons, which is then transmitted via the axons to the antennal lobes. The remaining odorant molecules are subsequently degraded partly by odorant-degrading enzymes (ODEs). Finally, the electrical signal is transmitted via projection neurons (PNs) of the glomeruli in the antennal lobes to higher-order olfactory centers, the mushroom body (MB) and the lateral horn (LH), ultimately triggering an appropriate behavioral response [2, 13–18].

Notably, in addition to being degraded by ODEs, odorant molecules can also be buffered by OBPs themselves through another mechanism where they titrate the release of odorants to modulate signal duration and intensity. The Carlson lab demonstrated this in Drosophila, where OBP28a-an abundant OBP restricted to ab8 sensilla-does not alter the response of olfactory receptor neurons, but acts as a buffer to stabilize odorant availability in the sensillar lymph [19, 20]. This buffering effect prevents rapid fluctuations in odorant concentration, ensuring consistent activation of downstream receptors and maintaining signal fidelity under fluctuating environmental conditions. The role of OBPs is discussed in a later section of this review.

Olfactory signaling in insects relies on combinatorial coding principle to encode diverse odorants, where a single odorant can activate multiple ORs, and individual ORs can respond to multiple odorants. This combinatorial coding expands the olfactory system’s capacity to discriminate a vast array of volatile compounds. For example, in Drosophila, the fruit volatile ethyl acetate activates a unique combination of ORs (OR42b, OR59b, and OR85a), while pentyl acetate activates a partially overlapping but distinct set of ORs [21, 22]. The insect brain decodes these combinatorial activation patterns in the antennal lobe, where each OR’s axons project to specific glomeruli, and the spatial and temporal integration of glomerular activity generates a unique odor fingerprint [23, 24]. This principle contrasts with the “labeled lines” mechanism [25] where a single OR responds to a specific ligand (e.g. pheromone receptor OR67d for cVA) [26], and together, these two strategies enable insects to both detect specialized signals (e.g., pheromones) and discriminate complex environmental odorants (e.g. plant volatiles).

Throughout the signal transduction process, multiple olfactory proteins are involved in recognizing and processing odorant molecules, including olfactory binding proteins, ORs, sensory neuron membrane proteins and ODEs [2]. In particular, olfactory binding proteins play a fundamental role in insect olfactory system and can be classified into three main groups: odorant-binding proteins (OBPs), chemosensory proteins (CSPs), and Niemann-Pick type C2 proteins (NPC2) [2, 4, 27, 28].

The first OBP was discovered and identified in the antennae of *Antheraea polyphemus* in 1981, and similar proteins have been continually identified in subsequent studies across numerous insect species [7, 29–31]. Advances in biotechnology, omics approaches, and bioinformatics have significantly accelerated research on olfactory binding proteins in insects, leading to an extensive accumulation of data. To date, a large number of proteins involved in olfactory binding have been identified in insects from orders Lepidoptera, Coleoptera, Hymenoptera, Diptera, and Hemiptera, Blattodea, Orthoptera, Thysanoptera [2, 13, 27, 32–43], with many of their biochemical functions and applications being extensively studied [8, 32, 44, 45], knowledge about this subject has been constantly increasing and updated. This review summarizes up-to-date progresses of the molecular characteristics, structure, function, and applications of insect olfactory binding proteins, providing insights and references for future research in this field.

## Molecular characteristics and structure of insect olfactory binding proteins

Insect olfactory binding proteins are a class of soluble, hydrophilic and low-molecular-mass proteins of about 15 kDa in size, and generally acidic. The N-terminal region contains a signal peptide composed of a dozen amino acids, which facilitates secretion into the extracellular space, where the protein performs its function. Structurally, these proteins adopt a spherical conformation, enabling them to bind a variety of ligand molecules [8, 46–48].

### Odorant-binding proteins (OBPs)

**Fig. 2:**
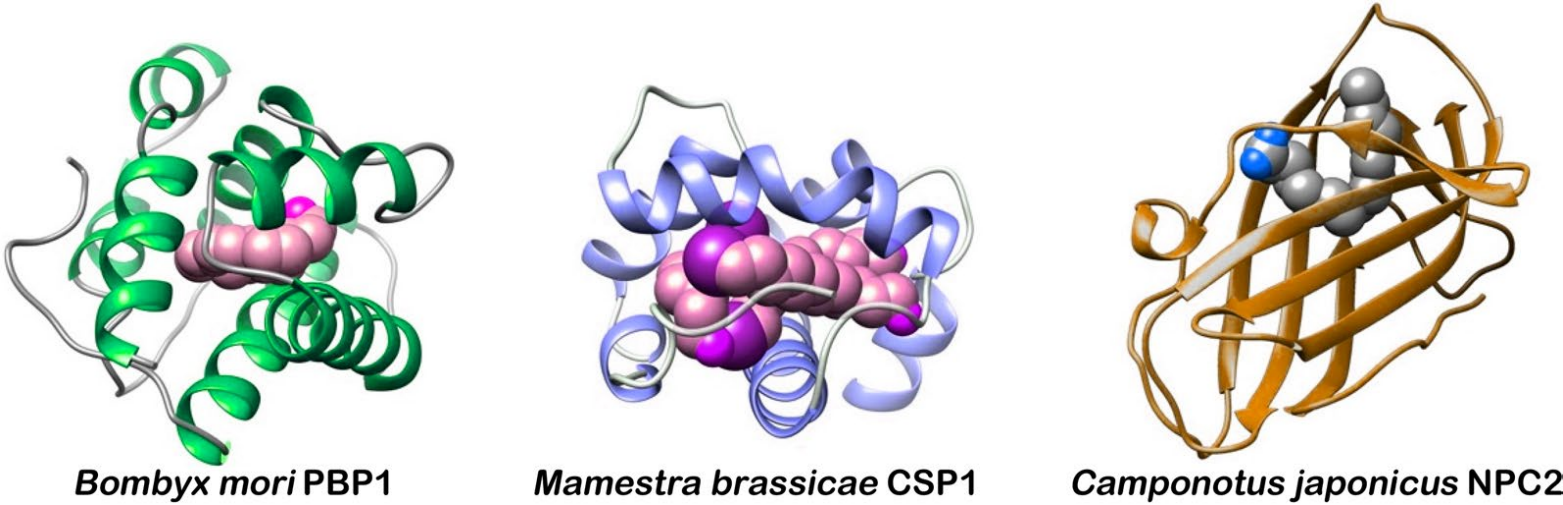
Schematic view of three-dimensional structures of *Bombyx mori* PBP1 [55], *Mamestra brassicae* CSP1 [32], and *Camponotus japonicus* NPC2 [56]. The three binding proteins are complexed with a ligand molecule in the internal binding cavity. While for CjapNPC2 with β-sheet secondary structures, BmorOBPs and MbraCSPs are primarily constituted by α-helical domains with a variable number of conserved cysteine residues. Despite the marked differences in structure, the three classes of proteins play similar roles in recognizing, binding, and transporting signaling chemicals in olfactory process, and are extremely compact and stable

The insect OBP family, one of the earliest identified groups of olfactory binding proteins, is characterized by its numerous members and structural complexity. Typical structural features include an abundance of α-helices and conserved cysteine residues [13, 49] (Fig. 2). Functionally, OBPs have been historically classified into three categories: general odorant-binding proteins (GOBPs), which bind common odorant molecules; pheromone-binding proteins (PBPs), involved in sex pheromone detection; and antennae-specific proteins (ASPs), localized primarily in antennae [50]. Initially, these classes were thought to have distinct roles, but accumulating evidence indicates that GOBPs, PBPs, and ASPs all exhibit broad ligand recognition capabilities [50–52]. A single OBP can bind diverse ligands-including general odorants and pheromones-while a single ligand may be recognized by multiple OBPs. Functional specificity thus depends on binding preference rather than strict categorical boundaries. Consequently, the traditional classification system presents limitations [53, 54].

OBPs can also be classified as classical, dimer, plus-C, minus-C, and atypical OBPs based on the number of conserved cysteine residues in their peptide chains [46]. Classical OBPs contain a typical six-conserved-cysteine (C1–C6) signature. Dimer OBPs feature two six-cysteine signatures. Plus-C OBPs contain two or three additional conserved cysteine residues along with one highly conserved proline residue. The extra cysteine residues are located immediately after the fourth and sixth cysteines, while the proline residue follows the sixth cysteine. Minus-C OBPs lack two conserved cysteines, typically the second and fifth. Atypical OBPs contain 9–10 conserved cysteine residues and have an extended C-terminal region [13, 29, 31]. The number of conserved cysteine residues determines the number of disulfide bonds within the OBP molecule, which in turn affects the protein’s structural stability. Due to their structural and physicochemical properties, OBPs perform several critical functions. Their hydrophilicity facilitates ligand (e.g. odorant molecule) binding, assisting in the transport of these ligand molecules to ORs. Their specific structural conformations enable the recognition and binding of diverse odorant molecules, leading to receptor activation [48]. Additionally, OBPs can modulate the persistence of odorant signals, ensuring a balanced and sustained olfactory response through their triple role in odorant transport, stabilization, and buffering changes in the odor environment (e.g. removing odorants from receptors or from the sensillar lymph). While ODEs primarily function to terminate signals by breaking down odorants, OBPs can slow this process by sequestering odorants in a bound state, reducing their accessibility to ODEs. This transient sequestration prolongs the availability of odorants for interaction with ORs, balancing rapid signal termination with sustained sensory input [7, 19, 33]. These characteristics underscore the importance of OBPs in insect olfaction, as they play a crucial role in detecting, processing, and responding to environmental chemical signals.

To date, the three-dimensional structures of more than 20 insect OBPs have been elucidated using X-ray crystallography and nuclear magnetic resonance (NMR) techniques. These structural data are available in the Protein Data Bank (PDB). All analyzed OBPs exhibit a spherical structure composed of α-helices, a feature that underlies their ability to bind diverse odorant molecules and fulfill their functions in the insect olfactory system [44, 46, 57].

### Chemosensory proteins (CSPs)

CSPs, another type of olfactory binding proteins discovered in insects, are a class of small, soluble, spherical proteins abundantly present in the sensillar hemolymph [58]. CSP genes were first identified in the vinegar fly *Drosophila melanogaster* in 1994, initially under the names Os-D and A-10 [59, 60], though their chemosensory function remained unknown at the time. It was not until 1999 that a homologous protein in the desert locust *Schistocerca gregaria* was confirmed to bind pheromones, leading to its designation as a CSP [9, 47]. CSPs are generally smaller than OBPs, typically ranging from 10 to 15 kDa, with an average mass of 13 kDa. Like OBPs, they consist of α-helices and contain conserved cysteine residues (Fig. 2). Most insect CSPs form six α-helices, though some have five or seven [61–63]. These variations in length and conformation allow CSPs to bind a wider range of ligands. A characteristic feature is the presence of four conserved cysteines forming two disulfide bonds (C1-X6-8-C2-X16-21-C3-X2-C4), which stabilize the tertiary structure [64, 65] and expand the ligand-binding pocket, enabling interaction with larger molecules [16, 62, 66]. CSPs exhibit higher evolutionary conservation than OBPs, with 40–50% cross-species sequence similarity, which may account for their smaller gene family size [61]. CSP genes are often tightly clustered in genomes, suggesting origin from a common ancestor. However, CSP numbers vary considerably across species, for instance, *Apis mellifera ligustica* has six, while *Bombyx mori* has 22 [66, 68]. Several CSP structures have been resolved to date, including those from the cabbage moth *Mamestra brassicae*, desert locust, and silkworm [64, 69, 70].

### Niemann-Pick type C2 proteins (NPC2)

NPC2 was first identified in vertebrates, where it plays a crucial role in cholesterol and lipid transport and is associated with disorders related to cholesterol metabolism [71]. Later researchers also discovered the presence of NPC2 proteins in arthropods, with Chelicerata serving as the most representative group. Insect NPC2 proteins exhibit high functional similarities to OBPs [27, 72], making them the third class of olfactory binding proteins in insects [48]. So far, the number of known insect NPC2 proteins is significantly lower than that of OBPs and CSPs, with 94 proteins identified across 19 species of 16 families from orders Orthoptera, Hemiptera, Coleoptera, Lepidoptera, Hymenoptera, and Diptera (Table 1). Evolutionary analyses suggest that NPC2 is a soluble protein that evolved earlier in arthropods than OBPs and CSPs [73]. Unlike OBPs and CSPs, primarily composed of α-helical secondary structures, insect NPC2 proteins predominantly feature β-sheet secondary structures (Fig. 2), forming a larger internal binding cavity [72]. Similar to OBPs and CSPs, NPC2 proteins contain conserved cysteine residues, typically four to six, which forms two to three disulfide bonds that stabilize their tertiary structures [28]. To date, the three-dimensional structure of an insect NPC2 protein has only been resolved in Japanese carpenter ant (*Camponotus japonicus*) [72].

**Table 1:** Insect species examined in this work with the number of NPC2 so far detected

Order	Family	Species	Number of NPC2s	Ref
Orthoptera	Oedipodidae	*Locusta migratoria*	2	[27]
Hemiptera	Aphididae	*Acyrthosiphon pisum*	2	[27]
	Miridae	*Cyrtorhinus lividipennis*	1	[36]
Coleoptera	Tenebrionidae	*Tribolium castaneum*	9	[27]
	Anobiidae	*Lasioderma serricorne*	2	[37]
Lepidoptera	Bombycidae	*Bombyx mori*	8	[27]
	Saturniidae	*Antheraea pernyi*	1	[38]
	Noctuidae	*Helicoverpa armigera*	1	[28]
Hymenoptera	Apidae	*Apis mellifera*	5	[27]
		*Apis cerana cerana*	4	[39]
	Megachilidae	*Megachile rotundata*	4	[27]
	Pteromalidae	*Nasonia vitripennis*	5	[27]
	Formicidae	*Camponotus japonicus*	1	[72]
	Eulophidae	*Baryscapus dioryctriae*	1	[40]
	Braconidae	*Microplitis mediator*	2	[41]
Diptera	Drosophilidae	*Drosophila melanogaster*	7	[27]
	Culicidae	*Culex quinquefasciatus*	13	[27]
		*Anopheles gambiae*	6	[27]
		*Aedes aegypti*	20	[42]

At the primary structure level, these proteins exhibit distinct characteristics in terms of length, conserved motifs, and post-translational modifications. Insect OBPs are generally the largest (~ 100–160 residues), followed by NPC2 (~ 130 residues) and the more compact CSPs (~ 100–120 residues) [74, 75]. A critical divergence is in glycosylation. NPC2 is uniquely N-glycosylated at specific sites (e.g. Asn-39 and variably Asn-116), a modification crucial for its stability, whereas OBPs and CSPs are typically non-glycosylated [76]. This structural divergence leads to the difference in tertiary fold. OBPs and CSPs share a similar “cupin fold” comprised of a β-barrel that forms a hydrophobic binding pocket, with OBPs often exhibiting pocket expansion and CSPs displaying a narrower entrance and significant flexibility [74, 77]. In stark contrast, NPC2 adopts a completely different β-sandwich structure with a well-defined hydrophobic cavity [78]. Consequently, properties of their ligand-binding pockets vary. OBPs possess the largest and most flexible pockets (~ 100–500 Å^3^), CSPs have smaller and relatively rigid ones (~ 50–200 Å^3^), and NPC2 features an intermediate-sized pocket (~ 200–300 Å^3^) with higher polarity due to key residues like Tyr100 that are essential for coordinating specific ligands like cholesterol [74].

Ligand-binding specificity varies considerably across these proteins. OBPs primarily bind volatile odorants like terpenes and aldehydes, as well as pheromones, with specificity dictated by the shape of their binding pocket. For example, rodent OBPs exhibit binding specificity for fatty acids, while insect OBPs show a strong preference for pheromone alcohols. In contrast, CSPs interact with a broader spectrum of ligands, including non-volatile lipids such as fatty acids and phospholipids, as well as environmental toxins; they show high affinity for medium-chain fatty acids, a preference attributed to their compact pocket structure, and in social insects like ants, CSPs are proposed to facilitate recognition of cuticular hydrocarbon signatures [8, 74]. NPC2, however, specifically accommodates sterol molecules like cholesterol and oxysterols, mediated by a conserved Tyr-Phe pair within its cavity [79]. It demonstrates strict selectivity for sterol moieties and does not bind typical OBP or CSP ligands such as glycolipids, phospholipids, or fatty acids, underscoring its unique functional role [76].

Insect OBPs and CSPs primarily function as extracellular carriers that deliver ligands to G protein-coupled receptors (GPCRs) in chemosensory tissues [2, 19], but CSPs also exhibit unique roles in non-chemosensory contexts, such as modulating inflammation by interacting with immune cell receptors [27, 58]. In contrast, NPC2 operates as an intracellular and extracellular cholesterol transporter, mediating transfer to NPC1 in endosomes to maintain cholesterol homeostasis and indirectly influencing signaling pathways such as SREBP via cholesterol levels, without engaging in direct GPCR signaling [71].

Although these proteins function as molecular buffers, their operational contexts and ligand specificity differ fundamentally. OBPs and CSPs act as extracellular buffers, solubilizing hydrophobic odorants and lipids in aqueous environments like mucus or hemolymph to prevent degradation and ensure efficient delivery to sensory receptors [58]. In contrast, NPC2 serves as a crucial intracellular buffer within the acidic environment of endosomes, where it maintains soluble cholesterol pools to prevent toxic crystallization and facilitates its safe trafficking to organelles like the ER and plasma membrane [71].

## Factors affecting the structure and function of insect olfactory binding proteins

**Fig. 3:**
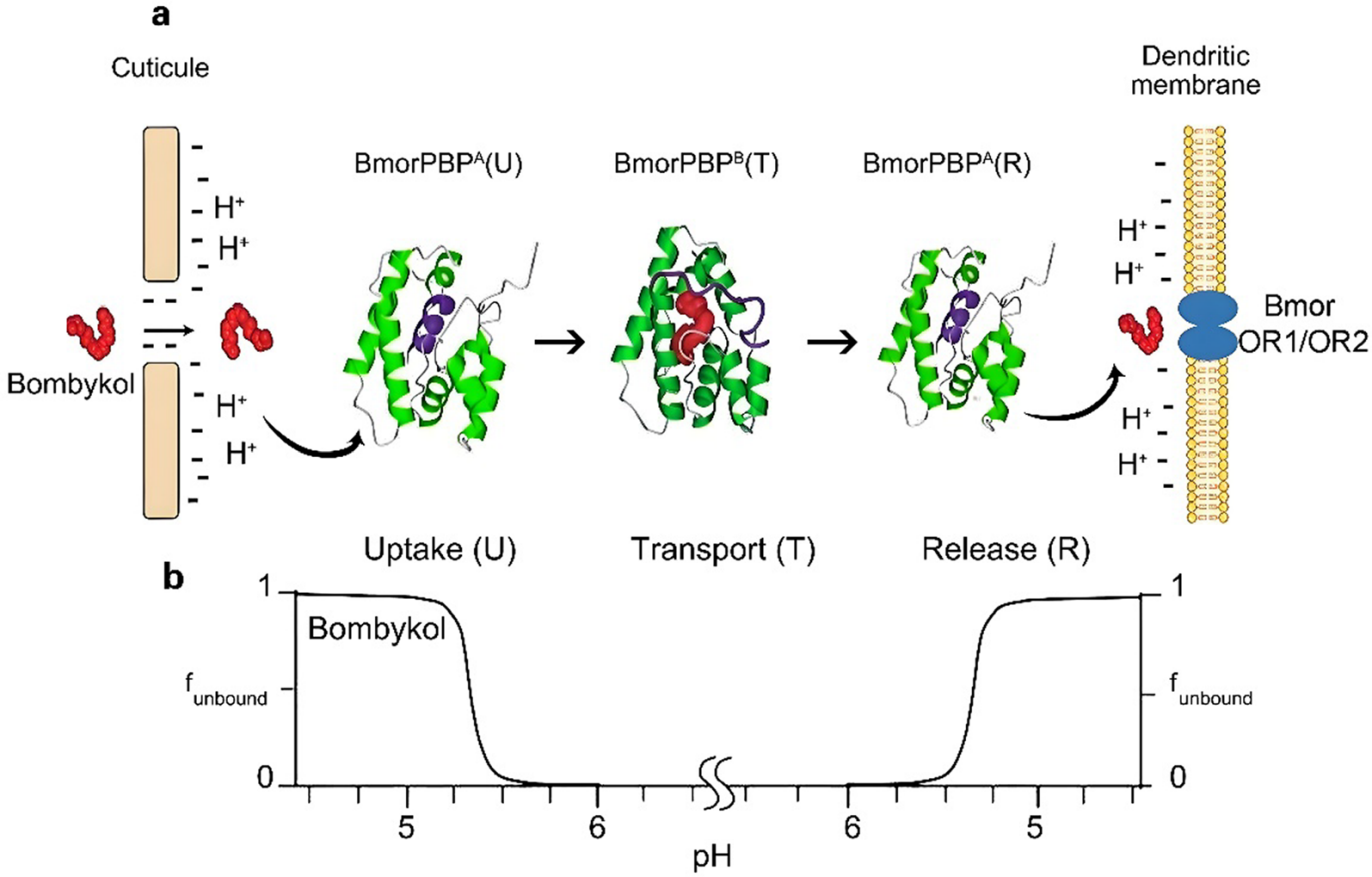
Model of *B. mori* pheromone transport mediated by pH-induced structural plasticity of BmorPBP [90]. **a** Process of *B. mori* pheromone transport. This process involves a sequence of coordinated steps. (1) Uptake and binding: Bombykol enters the sensillum through pores and binds to BmorPBP^A^ in the mildly acidic environment near the pores. (2) Conformational Change: Ligand binding induces a structural shift, converting the protein to BmorPBP^B^ state in a relative high pH environment. (3) Transport: The BmorPBP.^B^-bombykol complex diffuses through the sensillum lymph to the neuronal membrane in an environment with relatively stable pH. (4) Release: The acidic milieu near the membrane reduces the stability of the complex, triggering the release of bombykol to the ORs. **b** The pH gradient within the sensillum lymph regulates pheromone release, as shown by the increasing fraction of free bombykol at lower pH values. Adapted from [91]

The amino acid sequence directly influences the structure and function of insect olfactory binding proteins. Additionally, several other factors contribute to shaping the structure and function of these proteins. Firstly, environmental pH plays a crucial role. Studies have shown that the structure and ligand-binding affinity of Italian bee (*A. mellifera ligustica*) OBP1 (AmelOBP1) vary at different pH levels (4.0, 5.5, and 7.0) in vitro. At lower pH, AmelOBP1 adopts a conformation that enhances its binding affinity for the queen pheromone (E)-9-oxo-2-decenoic acid [80]. Interestingly, this phenomenon contrasts with the findings in the domesticated silkworm (*B. mori*) pheromone-binding protein (BmorPBP) and Gambian mosquito (*Anopheles gambiae*) OBP1 (*Agam*OBP1), which exhibit stronger ligand-binding affinities at higher pH levels [81, 82]. Once an OBP-bound ligand reaches the receptor, pH changes can also modulate OBP conformation to facilitate ligand release and receptor activation (Fig. 3). This pH-dependent regulation in body fluid is a critical mechanism in the function of insect olfactory binding protein [83]. Research reveals that pH of the sensillum lymph surrounding the olfactory neurons is not universal and can be influenced by insect physiology and diet [84, 85]. For example, blood-feeding insects like mosquitoes operate at a more neutral to slightly basic pH, which aligns with the pH of blood. In contrast, fruit-feeding Drosophila species may encounter a more acidic environment due to fermenting fruits. The optimal pH for their respective OBPs likely reflects an adaptation to their specific olfactory environment [32] Secondly, ligand binding itself induces the structural changes in some proteins. For example, AmelOBP14 undergoes conformational adjustments upon interacting with ligands such as 1-NPN, eugenol, lemonile, and tantalum bromide. These structural shifts enable proteins to accommodate various ligand molecules [86]. Given that OBPs often occur as dimers, the binding of one ligand to OBP may influence the binding dynamics of another [87]. Thirdly, amino acid mutations at specific sites can significantly impact the structures and functions of olfactory binding proteins. Such mutations may alter the protein’s conformation, affecting its ability to bind ligands. Mutations at key sites, particularly conserved cysteine residues and amino acids involved in the formation of hydrogen bonding, can disrupt protein–ligand interactions and impair olfactory signaling [10, 51, 86]. Studies suggest that mutants of olfactory binding proteins are found only in specific insect tissues, suggesting that these mutations may be associated with specialized functions [88]. For instance, site-directed mutagenesis experiments on *Chrysopa pallens* OBP4 (CPalOBP4) revealed that key amino acid substitutions affected its binding affinity for ligands such as farnesol [89]. Lastly, temperature influences the ligand-binding affinity of olfactory binding proteins. In vitro binding assays have shown that the binding affinities of Italian bee OBPs and CSPs for β-ocimene and alloocimene are temperature-dependent [54]. However, it remains unclear whether similar temperature effects occur within the insect body, as no in vivo studies have yet been reported.

## Action mechanism of insect olfactory binding proteins

Research on the action mechanisms of insect olfactory binding proteins, including ligand recognition, binding, transport, and release, remains incomplete and somewhat controversial [48]. The traditional mechanism holds that exogenous hydrophobic odorants rely on recognition and transport by olfactory binding proteins to reach ORs [92]. In addition to ligand transport, olfactory binding proteins may be also responsible for direct activity modulation of OR [93]. Currently, two main hypotheses regarding the mechanism by which olfactory binding proteins bind or release ligand molecule have been proposed: the pH-dependent regulation and the ligand-induced conformational change mechanism. Among these, the pH-dependent regulation is the prevailing hypothesis. As discussed earlier, olfactory binding proteins exhibit different ligand-binding affinities at varying pH levels, with lower pH typically reducing binding affinity, which suggests that pH fluctuations influence both ligand binding and release [2, 80, 94], (Fig. 3). Further research on this subject speculated that the pH within the antennal lymph is not uniform but instead exhibits regular change, potentially regulating the transport of the protein–ligand complex. However, this hypothesis has not yet been confirmed in insect antennae [84]. If the spatiotemporal variations of pH in antennal lymph could be demonstrated using physical and chemical techniques, it would significantly enhance our understanding of the action mechanism of the proteins. An alternative hypothesis proposes that the olfactory binding proteins function through ligand-induced conformational changes. The hydrophobic binding pocket within an olfactory binding protein determines its ligand-binding ability and its (more or less) broad specificity for odorants and pheromones. Ligand binding, in turn, induces structural changes in the proteins, facilitating more effective ligand recognition, delivery to receptors, and targetable receptor activation due to the structural flexibility [26]. This conformational shift not only enhances ligand targeting and receptor activation but also protects odorant and pheromone molecules from degradation by ODEs or pheromone-degrading enzymes (PDEs) in the lymph [48]. This protective role is essential for maintaining the normal functioning of the olfactory system in insects.

Although the traditional model posits that insect olfactory binding proteins recognize and ferry hydrophobic odorants to olfactory receptors, emerging evidence challenges this. ORs respond to ligands in vitro without OBPs in heterologous system, suggesting that some exogenous ligand molecules may reach ORs without OBP assistance. In vinegar fly, for example, knockout of the high-abundance DmelOBP genes did not eliminate antennal sensillum responses to exogenous odorant signals [85]. Deletion of sole highly expressed Obp28a in Drosophila ab8 sensilla enhanced electrophysiological responses to multiple odorants rather than impairing them [92] suggesting it may function as a concentration buffer [20] rather than an essential ligand transporter. Structural studies provide further support for this hypothesis, revealing that a large number of pore tubules (approximately 83,000 per sensillum) are distributed on insect olfactory sensilla, through which some exogenous molecules may directly diffuse to reach ORs without participation of olfactory binding proteins [11, 19]. Additionally, different sensilla within the same species may exhibit distinct structure. In vinegar fly, sensilla basiconica contain numerous pore tubules, whereas sensilla trichoidea lack the pores, but are instead enriched with OBP76a, suggesting that olfactory processing mechanisms may vary across different types of sensilla within the same insect species [95].

**Fig. 4:**
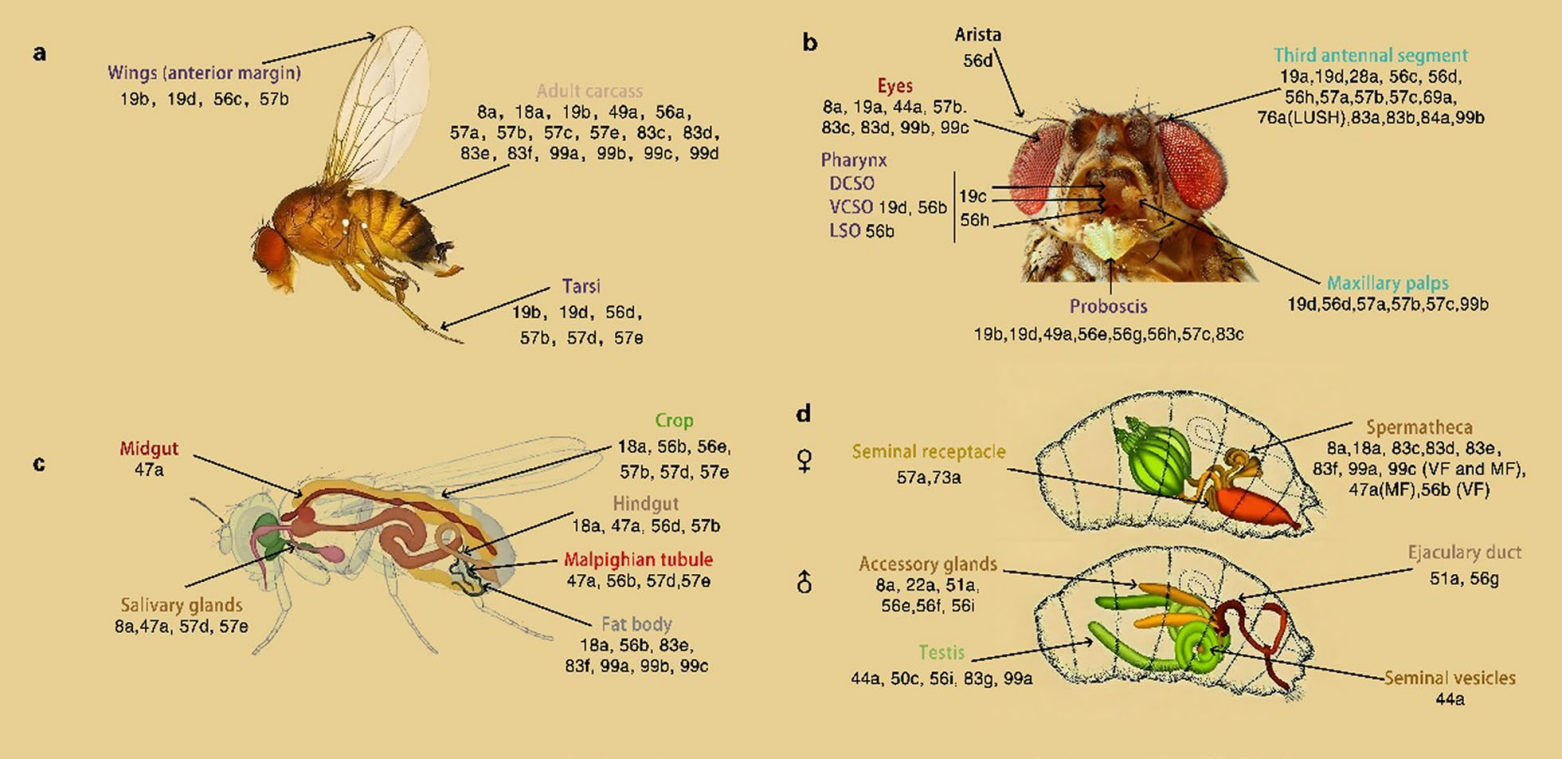
Expression and distribution of OBPs in chemosensory and non-chemosensory organs of *D. melanogaster*. OBPs expressed in each organ are represented by their number. **a** OBPs expressed in adult motor organs (wings and legs) and carcass, with their sensory function in the anterior margin of wings, leg tarsi, and adult carcass. **b** OBPs expressed in adult head (eyes, antennae (the third antennal segment and arista), proboscis, maxillary palp and pharynx (the labral sense organ (LSO), the ventral sense organs (VCSO) and dorsal cibarial and (DCSO)). **c** OBPs expressed in adult digestive organ (midgut, hindgut, Malpighian tubule and crop), fat body, and salivary glands. **d** OBPs expressed in the reproductive organs of larva (female: seminal receptacle, spermatheca; male: accessory gland, testis, ejaculary duct, and seminal vesicle). Adapted from [90]

An increasing number of evidence indicates that olfactory binding proteins are extensively distributed not only in olfactory organs but also across non-olfactory organs in insects [96, 97], (Fig. 4). presenting more significant challenges for elucidating their functional roles and mechanisms of action outside the olfactory system. Research has shown that olfactory binding proteins can also be expressed in Drosophila taste sensilla [59, 60], (Fig. 4), particularly, some of them are involved in taste perception. OBP49a expressed in the *D. melanogaster* labellum inhibits sweet-sensing neurons upon biter compound binding, yet its mechanism-direct receptor or neuronal modulation-remains unverified. OBP57d and OBP57e expressed in *D. melanogaster* leg sensilla are involved in the oviposition response to C6–C9 fatty acids [90]. Additionally, in Drosophila, mutant flies lacking OBP59a exhibit reduced hygrotaxis, but OBP59a likely maintains structural integrity of sensilla rather than transporting water molecules. The broad expression of OBPs in non-olfactory organs suggests their functional differentiation into local signaling modulation, yet most mechanisms remain at the hypothetical level and require analysis by combining in vivo ligand identification with cell-specific knockout approaches [90].

## Expression and distribution of insect olfactory binding proteins

**Fig. 5:**
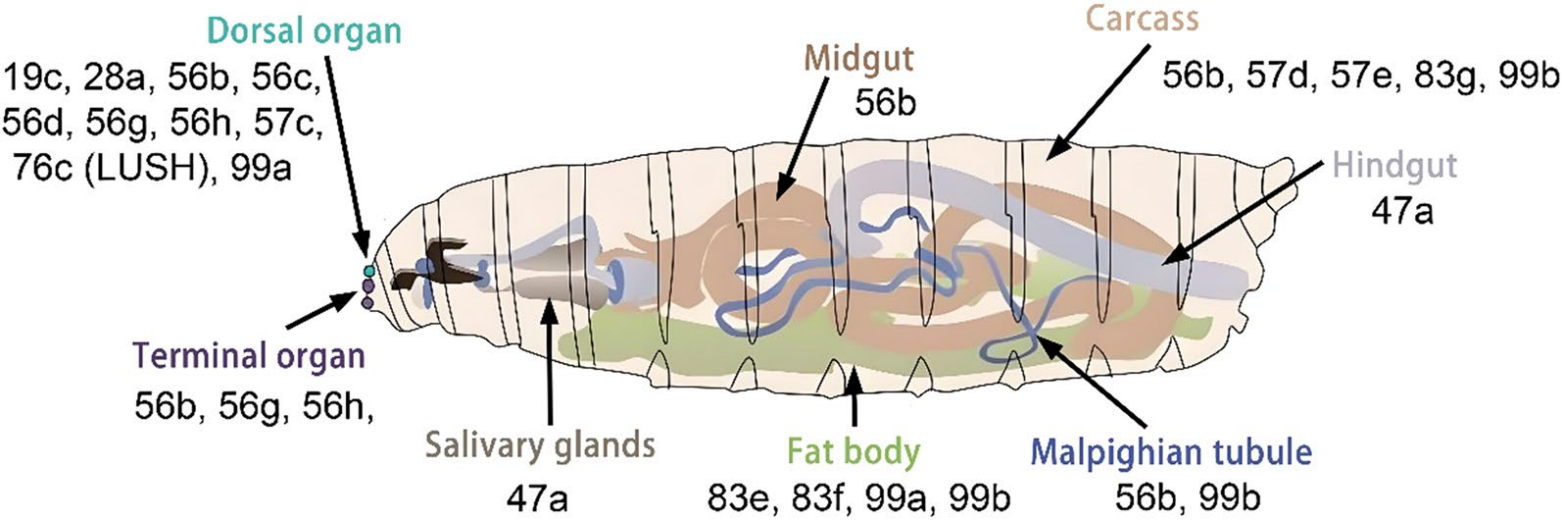
OBPs expression pattern in the olfactory, gustatory, digestive, excretory organs and certain tissues (fat body) and glands (salivary gland) of *D. melanogaster* larvae [90]

Early studies on insect olfactory binding proteins primarily focused on their expression and distribution in olfactory organs, where they are found in high concentrations within the lymph [96]. However, substantial evidence has revealed that OBPs are expressed in the majority of insect organs [97–102, 104] (Fig. 4). In insects, the first olfactory binding proteins identified in Hymenoptera and Lepidoptera, were OPBs. They are differentially or specifically expressed in males and females, and play a key role in recognizing pheromone molecules, such as AmelASP1 in the Italian bee [97] and AsegPBP in the turnip moth *Agrotis segetum* [98]. Later, during studies of insect behaviors related to parasitism and feeding, researchers discovered numerous GOBPs in the antennae, which are involved in detecting plant-derived odorants [7]. With further research, it became evident that OBPs are also expressed in tissues beyond insect antennae (Figs. 4, 5), [99–102]. In *Drosophila melanogaster*, for instance, OBPs are widely distributed across various insect stages: certain OBPs are expressed in both larvae and adults, whereas others are specific to larvae (Fig. 5). Most OBPs present in larvae exhibit similar expression patterns across adult tissues (Figs. 4 and 5). Moreover, the functions of OBPs expressed in different parts of larvae vary: OBPs expressed in larval dorsal organs show affinity for volatile odorants, whereas those expressed in terminal organs exhibit affinity for both volatile and soluble compounds [90]. The rapid advancement of high-throughput sequencing and proteomics has accelerated research on insect olfactory binding proteins, enabling more comprehensive and in-depth analyses [103]. Combined experimental and bioinformatics studies, for example, have identified 21 OBPs in the Italian bee, 13 of which are expressed in the antennae (including two that are antenna-specific). Other OBPs have been found in the brain, thorax, ovaries, and larvae [104]. Interestingly, some OBPs are exclusively expressed in non-olfactory organs, suggesting they serve specialized functions, which will be explored further in discussions of their functional roles (Fig. 4, Table 2).

**Table 2:** Putative functions of olfactory binding proteins in insects assessed from experiment, bioinformatics, and omics analysis

Putative function	Protein types	Species	Ref
Perception/avoidance of plant volatiles or pheromones	TabsOBP	*Tuta absoluta*	[4]
	PstaOBP1	*Plautia stali*	[49]
	AgerOBP23	*Apriona germari*	[102]
	GmolGOBP2/ GOBP3 GmolPBP2/PBP3	*Grapholitha molesta*	[105]
	LacuCSP7/11	*Leptocorisa acuta*	[106]
	DcitCSP1/12	*Diaphorina citri*	[107]
	AipsCSP2/8/9/10	*Agrotis ipsilon*	[108]
	PrapCSP16	*Pieris rapae*	[10]
	NlugCSP3/5/10	*Nilaparvata lugens*	[109, 110]
	SlitCSP3	*Spodoptera litura*	[111]
	RpadCSP4/5/6	*Rhopalosiphum padi*	[112]
	EbraCSP8	*Eucryptorrhynchus brandti*	[66]
	CforCSP1/5/6	*Cylas formicarius*	[9]
	CmedCSP33	*Cnaphalocrocis medinalis*	[113]
	PrapCSP6/10/11	*Pieris rapae*	[10]
	HarmNPC2-1	*Helicoverpa armigera*	[28]
	CjapNPC2	*Camponotus japonicus*	[72]
	McinNPC2	Macrocentrus cingulum	[114]
	BdioNPC2b	*Baryscapus dioryctriae*	[40]
	AcerNPC2	*Apis cerana cerana*	[39, 115]
	MmedNPC2	*Microplitis mediator*	[41]
Involvement in insect growth, development, reproduction, and other physiological functions	DmelOBP56f/OBP56g	*Drosophila melanogaster*	[116]
	GqinOBP2/OBP12/OBP17	*Gynaephora qinghaiensis*	[117]
	SfruOBP31	*Spodoptera frugiperda*	[118]
	AtumOBP1	*Aethina tumida*	[31]
	AligASP1	*Apis mellifera ligustica*	[97, 119]
	AmelCSP5	*Apis mellifera ligustica*	[120]
	AmelCSP4	*A. mellifera ligustica*	[121]
	CresCSP1/2/4	*Clostera.restitura*	[122]
	NlugCSP3/10	Nilaparvata lugens	[110]
	PxylCSP11	*Plutella xylostella*	[123]
	SsubCSP1/3/6/16	*Scopula subpunctaria*	[124]
	OcomCSP12	*Ophraella communa*	[125]
	PrapCSP7/16/18/20	*Pieris rapae*	[10]
	DmelPHK3	*Drosophila melanogaster*	[126]
	DmelPEBⅢ/DmelPHK3	*Drosophila melanogaster*	[127]
	NPC1a	*Drosophila*	[128]
	BmorNPC2	*Bombyx mori* L	[129]
	AaegNPC2	*Aedes aegypti*	[42]
	BdioNPC2	*Baryscapus dioryctriae*	[40]
Involvement in resistance to pesticides in insects	PverOBP18	Plagiodera versicolora	[130]
	BmorCSPs	*Bombyx mori*	[70]
	PxylCSP4/8	*Plutella xylostella*	[131]
	BtabCSP1/12	*Bemisia tabaci*	[132, 133]
	NlugCSP3	*Nilaparvata lugens*	[110]
	LmigCSP3	*Locusta migratoria manilensis*	[134]
	RpadCSP4/5/6/10	*Rhopalosiphum padi*	[112]
	CsinCSP2/3/5	*Conopomorpha sinensis*	[62]
Assist in activating the receptors	DmelOBP76a	*Drosophila melanogaster*	[26, 135]
Physiological buffering	DmelOBP28a	*Drosophila melanogaster*	[19, 20]
Release and transport of pheromones	HarmOBP10	*Helicoverpa armigera*	[136]
	AmelOBPs/CSPs	*Apis mellifera*	[120, 137]
	LhetOBPs/CSPs	*Leptopilina heterotoma*	[138]
	BmorCSPs	*Bombyx mori* L	[139]
	LmigCSP91	*Locusta migratoria*	[140, 141]
	PpupOBPs/CSPs	*Pteromalus puparum*	[142]
	AipsOBPs/CSPs	*Agrotis ipsilon*	[27]
	AaegOBPs/CSPs	*Aedes aegypti*	[27]
Tissue regeneration and repair	AaegOBP1/11/13/44/45	*Aedes aegypti*	[143, 144]
	AmelCSP5	*Apis mellifera ligustica*	[67, 145]
	SinvCSP9	*Solenopsis invicta*	[146]
	SexiCSP3	S*podoptera exigua*	[147]
	LmigCSPs	*Locusta migratoria*	[148]
	PameP10	*Periplaneta americana*	[149, 150]
	AAEL006854/AAEL020314	*Aedes aegypti*	[42]
Anti-inflammatory & immune activity	AsteOBPs	*Anopheles stephensi*	[151]
	SfruOBPs	*Spodoptera frugiperda*	[118]
	PxylOBP13/CSP4/CSP8	*Plutella xylostella*	[132]
	D7-related proteins	*Anopheles stephensi*	[152]
	D7-related proteins	*Phlebotomus argentipes*	[153]
	D7-related proteins	*Aedes aegypti*	[154]
	AcerNPC2a	*Apis cerana cerana*	[155]
	DmelNPC2a/NPC2e	*Drosophila melanogaster*	[156]
Taste and nutrition function	PregOBP57a	*Phormia regina*	[157]
	DmelOBP19b	*Drosophila melanogaster*	[158]
	AmelOBP4	*Apis mellifera*	[159]
	MbraCSPs	*Mamestra brassicae* L	[160]
	HarmCSP4	*Helicoverpa armigera*	[133]
	HassCSP4	*Helicoverpa assulta*	[133]
	MbraCSPA6	*Mamestra brassicae* L	[160]
	SgreCSP15	*Schistocerca gregaria*	[47]

Compared to OBPs, CSPs exhibit a broader distribution in insects. This is partly due to the widespread presence of sensilla with chemosensory functions across various body parts, including hairs, antennae, appendages, mouthparts, and wings [8]. Additionally, CSPs themselves possess greater functional diversity. In Italian bee, genome-wide analysis has identified six CSPs, of which CSP5 and CSP6 are not expressed in the antennae. Notably, CSP5 is specifically expressed in the queen’s ovaries and eggs, while the remaining CSPs are distributed across different tissues and developmental stages [67]. In silkworm, 22 CSPs have been identified, with expression observed in a wide range of tissues, including eggs, larvae of different ages, adult brains, epidermis, fat body, midgut, and reproductive organs (sperm and ovaries). The spatial and temporal variations in CSP expression highlight their diverse roles in insect development and physiology [68].

So far, research on NPC2 in insects remains limited. NPC2 family genes have been identified in only 6 orders Orthoptera, Hemiptera, Coleoptera, Lepidoptera, Hymenoptera and Diptera, spanning 16 families and 19 species, with 94 NPC2 genes reported to date (Table 1). Correspondingly, studies on their spatial and temporal expression in insects are scarce. Based on limited available data, NPC2 genes are expressed in both olfactory and non-olfactory organs, with functions that vary depending on the species and the specific organ in which they are expressed [48].

## Functions of olfactory binding proteins

The relatively simple yet stable structure of insect olfactory binding proteins enables them to adapt to diverse environments and serve various complex functions (Table 2), which are significantly crucial for the physiology and behavior of insects.

### Detection and transport of exogenous odorants and pheromone molecules

The discovery of olfactory binding protein function was initially in insect olfactory system, only highlighting their roles in the binding and transport of odorant and pheromone molecules. LUSH (DmelOBP76a) was the first functionally characterized OBP in the vinegar fly and is critical for pheromone detection [48]. In the Italian bee, AmelASP1 is required for transporting the queen pheromone HOB to OR11 [97, 119]. However, olfactory binding proteins are responsible for not only transporting molecules but also for recognizing different odorants and pheromone molecules. In *Clostera restitura* (a species of moth), CresCSP1, CresCSP2, and CresCSP4 are highly expressed in the 2nd to 4th instar larvae, suggesting their involvement in detecting host volatiles for food location [53]. Similarly, in the cabbage butterfly *Pieris rapae*, PrapCSP16 is primarily expressed in female antennae and may contribute to host-seeking and chemical communication behaviors [10]. In the parasitic wasp *Baryscapus dioryctriae*, NPC2 proteins also function as odorant carriers inside body [40]. Research has demonstrated that OBPs and CSPs exhibit broad ligand-binding specificity, though their affinities for different ligand molecules vary [54]. For example, in the Italian bee, most of OBPs and CSPs can bind larval pheromones such as β-ocimene and allo-ocimene. Among them, CSP4 has the highest binding affinity for both molecules and individuals with higher CSP4 expression show improved larval recognition—due to the enhanced ability to identify brood pheromones—and higher royal jelly production [121]. In tsetse fly *Glossina fuscipes fuscipes*, silencing GffOBPs with strong interactions with 1-octen-3-ol (a known tsetse attractant) significantly eliminated the flies’ attraction to this compound, demonstrating their direct involvement in olfaction [161].

Beyond the traditional role in olfaction, insect olfactory binding proteins have shown a diverse range of physiological functions in chemical communication, mediating detection, and integration of semiochemicals [43, 53, 97, 119]. They enable precise recognition and transport of conspecific pheromones (e.g. *Bombyx mori* BmorPBP1 for bombykol, *Drosophila melanogaster* LUSH for cVA) to ensure reproductive success. Additionally, OBPs facilitate adaptation to host-derived chemicals, such as *Anopheles gambiae* AgamOBP1 binding indole for host location [162] or *Drosophila sechellia* OBP57d/57e tolerating toxic plant volatiles [43]. OBPs such as *Cnaphalocrocis medinalis* CmedPBP4 and *Athetis lepigone* AlepGOBP2 also exhibit dual binding plasticity, linking pheromones and plant volatiles to coordinate foraging and mating in dynamic environments [43].

### Assisting in receptor activation

Olfactory binding proteins play a key role in facilitating the activation of insect ORs. Insect ORs function as heteromeric ligand-gated ion channels that, upon ligand binding, initiate a signaling cascade [2, 163]. In olfactory signal transduction, most ligand molecules directly activate ORs, while some require the involvement of olfactory binding proteins. In such cases, the protein-odorant complex is essential for receptor activation [2]. Studies have demonstrated that vinegar fly individuals lacking LUSH fail to respond to the pheromone Z11-18OAc, and neural stimulation only occurs in the presence of LUSH, indicating that LUSH is essential in assisting in receptor activation by Z11-18OAc [135]. Further research revealed that the male-specific pheromone component, cis-vaccenyl acetate, also requires complex formation with LUSH for receptor activation [26]. To date, LUSH remains the only OBP reported to assist in receptor activation, with no similar findings in other insects.

### Buffering effect

The buffering of olfactory binding proteins inside the sensillar hemolymph plays a significant role in insect physiology. By binding to specific molecules, they help regulate their local concentrations and mitigate physiological fluctuations. Some insect olfactory binding proteins, despite being highly expressed in olfactory organs, do not participate in odorant transport but instead function as buffers [32]. In vinegar fly, DmelOBP28a is the most abundantly expressed OBP in the antennal sensilla basiconica. However, its knockout does not affect olfactory responses, suggesting that it serves a buffering function, acting as a “gain control” mechanism [19]. Further studies have shown that DmelOBP28a buffers various plant-derived odorant molecules within the antennae, such as β-ionone, preventing continuous stimulation from these molecules [20].

### Transport and release of endogenous pheromones

Insects possess various glands containing numerous olfactory binding proteins that are highly or specifically expressed and facilitate the transport and release of pheromones in the glands, playing a crucial role in insect physiology and ecology. Insect pheromones are highly diverse and serve as essential mediators of both intra- and interspecific chemical communication [164]. Proteomic analyses have identified seven CSPs specifically expressed in the female gonads of domesticated silkworm [139], likely involved in the transport and release of sex pheromones. In the vinegar fly, six OBPs are found in male semen, where they bind to the male-specific pheromone cis-vaccenyl acetate [165, 166]. Similarly, in cotton bollworm *Helicoverpa armigera*, HarmOBP10 is highly abundant in male semen and is transferred into the female body during mating, ultimately remaining on the surface of fertilized eggs [136]. In the East Asian locust *Locusta migratoria*, various CSPs are present in male genitalia, with highly expressed LmigCSP91 effectively binding α-naphthyl propionitrile and β-naphthyl propionitrile [140, 141]. Beyond reproductive glands and gonads, OBPs and CSPs are also found in the poison glands of Hymenopteran insects. For instance, in parasitic wasps (*Leptopilina heterotoma* and *Pteromalus puparum*), OBPs and CSPs are present in poison glands, suggesting that both proteins play a potential role in venom secretion and release [138, 142]. Proteomic analyses have also revealed the presence of OBP21 in the poison glands of Italian bee, though its function remains unclear [167]. Additionally, nine OBPs and two CSPs have been detected in the mandibular glands of honeybees [120, 137], potentially contributing to pheromone secretion and release in these glands. Western blot analysis has revealed the presence of a large number of CSP mutants in silkworm pheromone glands, indicating their potential ability to bind a broad range of ligands [88]. OBPs and CSPs have also been identified in the glands of insects such as the cabbage moth, black cutworm (*Agrotis ipsilon*), and Egyptian mosquito (*Aedes aegypti*), though their specific functions remain to be elucidated [27].

### Development, repair, and tissue regeneration

Interestingly, OBPs and CSPs also play important roles in insect development and even tissue regeneration. One of the earliest observations of olfactory binding proteins involvement in insect development was in American cockroach *Periplaneta americana*, where protein immunoblotting revealed a significant upregulation of PameP10 expression during leg regeneration [150], with expression level increasing to 30 times the normal level [149]. Transcriptomic analysis in Italian bee indicated that AmelCSP5 is specifically expressed in the ovaries and eggs, suggesting a potential role in embryonic development [67, 145]. Similarly, in the fire ant (*Solenopsis invicta*), SinvCSP9 is highly expressed in 3-day-old larvae and is involved in cuticle formation and ecdysis by influencing fatty acid biosynthesis and other metabolic pathways [146]. In Egyptian mosquito, several OBPs, including AaegOBP1, AaegOBP11, AaegOBP13, AaegOBP44, and AaegOBP45, are thought to contribute to egg membrane formation, although their exact regulatory mechanisms remain unclear [143, 144]. In the beet armyworm (*Spodoptera exigua*), SexiCSP3 is expressed at higher levels in female reproductive organs than in males. RNAi targeting this protein inhibits egg-laying and significantly reduces hatching rates, highlighting its essential role in egg formation and development [147].

Sterols are crucial for insect growth and development. However, since insects cannot synthesize them themselves, they must obtain the substances from their diet [168, 169]. Studies have identified four AaegNPC2 proteins in Egyptian mosquito as potential sterol-binding proteins. Among these, AAEL006854 and AAEL020314 may play key roles in ecdysteroid biosynthesis and molting, while another NPC2 protein is suspected to function as a potential fatty acid-binding protein [42].

### Anti-inflammatory and immune activity

The anti-inflammatory and immune functions of insect olfactory binding proteins were first identified in hematophagous insects [48]. When mosquitoes bite a host, the resulting immune response leads to blood coagulation, which can interrupt blood sucking. Studies have found that certain OBPs in the saliva of *Anopheles stephensi* play an anticoagulant role, preventing bloodsucking disruption [151]. Additionally, during blood sucking, the host secretes biogenic amines such as norepinephrine and serotonin, which can cause redness, swelling, and itching. OBPs in the saliva of Gambian mosquitoes and Egyptian mosquito exhibit a strong affinity for these biogenic amines, helping to suppress the host’s inflammatory response and facilitating uninterrupted feeding [118, 170, 171]. In the vinegar fly, DmelPEBⅢ and DmelPHK3 are thought to contribute to immune defense against bacterial and viral infections, playing roles in tissue repair and pathogen recognition [172]. Similarly, in the Gambian mosquito, the transcription of AgamIR7 increases significantly after exposure to bacterial lipopolysaccharide (LPS) for six hours. The strong homology between DmelPEBⅢ and AgamIR7 [173] suggests they share similar immune functions. Further research in the vinegar fly has shown that overexpression of NPC2a and NPC2e can activate innate immunity via the *Diptericin* promoter [156]. Additionally, D7-related proteins in the Egyptian mosquito and sandfly (*Phlebotomus argentipes)* share structural similarities with OBPs and both exhibit immunogenic properties [153, 154]. In the Chinese honeybee (*Apis cerana cerana*), AcNPC2a plays a key role in stimulating innate immunity during early bacterial and fungal infections [155]. In the Egyptian mosquito, NPC2 has been shown to significantly reduce dengue virus infection [174]. In locusts, the volatile compound phenylethyl alcohol (PEA) secreted from *Metarhizium anisopliae* (Green muscardine fungus) causes immune responses due the presence of OBP11, indicating that locust OBP11 play a crucial role in pathogen immune [175]. While these proteins display anti-inflammatory and immune activity, the underlying signaling pathways and regulatory processes remain poorly understood, requiring further research to clarify their role in the immunity response.

### Pesticide and pathogen resistance

Resistance to pesticide and pathogen is a major challenge in insect pest control, with olfactory binding proteins potentially playing a role in its development. In response to pesticide or pathogen exposure, insects mount a complex physiological defense, with OBPs and CSPs being significantly upregulated as part of this resistance mechanism. This was first demonstrated in silkworm larvae, where insecticide treatment triggered a marked increase in BmorCSPs expression [70]. This physiological response is conserved across insect species. Whiteflies *Bemisia tabaci* exhibited elevated BtabCSP1 levels in abdominal tissues after sub-lethal neonicotinoid exposure [176], and silkworms responded to abamectin by upregulating 20 different CSPs in various tissues, with some proteins exhibiting a high affinity for abamectin [177]. Similarly, permethrin exposure induced the expression of PxylOBP13, PxylCSP4, and PxylCSP8 in diamondback moths *Plutella xylostella* [131]. In *Aphis gossypii*, the AgosCSP4/5/6*-*insecticide complex exhibited greater stability than the AgosCSP4/5/6*-*other compound complex, suggesting that AgosCSPs participate in resistance development. These findings provide new insights into the mechanisms of insecticide resistance mediated by CSPs [178]. Beyond chemicals, this response also extends to pathogens, as evidenced by at least 1.8-fold upregulation of PverOBP18 in *Plagiodera versicolora* following bacterial infection [130]. Physiologically, these proteins are believed to contribute to resistance either by directly binding and sequestering pesticide molecules or by initiating downstream signaling pathways that activate broader defensive processes. However, the precise physiological mechanisms through which these proteins confer resistance remain an area of active investigation [32].

### Taste, feeding, and nutrient absorption

Insect olfactory binding proteins are highly expressed in insect taste organs and may play essential roles in feeding and nutrient absorption [31, 65]. Firstly, one key function of insect olfactory binding proteins is facilitating the uptake of hydrophobic substances. For example, in the black blowfly (*Phormia regina*), which prefers carcasses enriched with water-insoluble fatty acids, PregOBP57a expressed in the oral disk is thought to aid in the uptake of hydrophobic fatty acids by altering pH [157]. In cotton bollworm, HarmCSP4 is highly expressed in the mouthparts, where it binds to hydrophobic compounds such as β-carotene, assisting in the absorption of essential nutrients [179]. Secondly, this type of proteins also plays a role in the feeding process. In both the cotton bollworm and the tobacco hornworm (*Helicoverpa assulta*), CSP4 is highly expressed in the proboscis. During feeding, CSP4 is secreted to lower hydrostatic pressure, ensuring a smooth flow of plant sap into the proboscis [133]. Additionally, insect olfactory binding proteins exhibit high affinity for key nutrients. In vinegar fly, DmelOBP19b is widely distributed and binds rapidly to essential amino acids such as L-phenylalanine and L-glutamine, facilitating their transport [158]. Studies on blood-feeding insects suggest that their feeding behavior may be linked to the evolution of the CSP gene family, with these genetic adaptations influencing their dietary habits [180].

## Practical applications of olfactory binding proteins

### Insect pest management

Conventional pesticide-based approaches have been widely used in insect pest management, but their non-selective use have led to several negative consequences such as environment pollution, development of insecticide resistance, and harm to no-target organisms [175]. In recent years, there has been growing interest to shift the traditional pest management strategies to eco-friendly and sustainable ones. An emerging approach gaining attention in agriculture and forestry is the use of insect olfactory binding proteins, which play a crucial role in foraging, mate-seeking, and host detection [2, 52, 181, 182]. In this context, reverse chemical ecology approaches can facilitate the development of insect pest management strategies that contribute to a sustainable agricultural production model [183]. Insect pest management based on the insect olfactory system primarily involves three approaches: attraction, poisoning, and repulsion.

#### Attraction-based pest control

This approach suppresses pest populations by using insect pheromones or other odor-based attractants to lure insect pests, which reduces the reliance on chemical pesticides [184]. For example, *Plautia stali*, an agricultural pest, can be effectively attracted using a synthetic compound derived from its aggregation pheromone, methyl (E, E, Z)-2,4,6-decatrienoate, which can be incorporated into traps for pest capture and management [185]. Similarly, the pea aphid (*Acyrthosiphon pisum*), a widely distributed and serious legume pest, responds strongly to its sex pheromones, (4aS,7S,7aR)-nepetalactone and (1R,4aS,7S,7aR)-nepetalactol, which can be synthesized to lure aphids into traps, achieving the goal of controlling pest populations [186]. Another example is *Athetis lepigone* AlepGOBP2 with strong binding affinity for sex pheromones such as (Z)-7-dodecenyl acetate and (Z)-9-tetradecenyl acetate [187]. By synthesizing these pheromones or their analogs, pest-specific traps can be designed for effective control in the field.

Another attraction-based pest management strategy is to disrupt the normal insect behavior by interrupting the insect communication with the surrounding environments. The approaches such as genetic modifications, RNAi and mutation of OBPs have proven effective and species-specific in insect behaviors alteration [175]. In oriental fruit fly, adult males with BdorOBP56f-2 knocked out via in-vivo CRISPR/Cas9 showed significantly reduced attraction to females, confirming this protein’s necessity for methyl eugenol perception [188]. SinvOBP5 from the red imported fire ant, *Solenopsis invicta* exhibited high antennal expression. RNAi knockdown of SinvOBP5 significantly reduced electroantennography (EAG) responses to alarm pheromones and the alkaloid, 2,4,6-trimethylpyridine and [189]. Similarity, RNAi-mediated suppression of DcitOBP7 mRNA significantly decreased both EAG activity and adult *Diaphorina citri* behavioral reactions to the tested ligands [190]. Such gene editing technologies have provided deep insights into design of insect pest management approaches, demonstrating significant application potential in agricultural and forestry pest management.

#### Poisoning-based pest control

This approach disrupts insect growth and development by inhibiting or blocking olfactory signal transmission using specific chemicals, achieving the purpose of suppressing the pest populations [191]. Researchers design insecticidal molecules with high binding affinity to insect OBPs, thereby blocking normal olfactory signaling and impairing pest behavior and growth [32]. For instance, *Athetis lepigone* AlepGOBP1 has a strong affinity for insecticides such as chlorpyrifos and phoxim. By synthesizing these compounds or their molecular analogs used in pest control, the normal olfactory signaling mediated by AlepGOBP1 in *A. lepigone* can be disrupted, achieving an effective pest control [187]. Another example involves (E)-β-farnesene, an important alarm pheromone for the pea aphid. While it can be used for pest control, its volatility and susceptibility to oxidation pose challenges for practical application. To address this, molecular analogs of (E)-β-farnesene have been synthesized with a pyrazole structure, enhancing their stability and binding affinity to pea aphid OBPs while also exhibiting high insecticidal activity [192].

While insecticides can effectively reduce pest populations, they may also negatively impact beneficial insects. For instance, Chinese honeybee, a key pollinator in traditional Chinese agriculture, can be adversely affected by neonicotinoid insecticides such as imidacloprid. Imidacloprid has been shown to reduce the ability of Chinese honeybee ASP2 to recognize odorant molecules, potentially impairing its olfactory response [193]. Similar effects have been observed in the Chinese honeybee CSP1 [194] and tea geometrid (*Ectropis obliqua*) GOBP2 proteins [195]. Therefore, when designing insecticides, it is essential to balance efficacy in pest control with potential risks to beneficial insects. Enhancing pesticide specificity can help mitigate unintended ecological impacts while maintaining effective pest management strategies.

#### Repulsion-based pest control

Repulsion-based pest control is a widely used biological control method that relies on specific environmental stimuli to keep pests away, achieving the goal of protecting agricultural resources [196]. Currently, two primary theories explain the mechanisms behind repellent behavior: the “odorant-binding type” and the “contact type”. Insect repellent behavior is widely thought to occur when the olfactory system detects small molecular compounds, such as plant secondary metabolites (PSMs) or environmental odorants, triggering avoidance responses. This mechanism, known as the odorant-binding type [197], effectively explains the action of small, volatile molecules. However, research has shown that plant secondary metabolites with larger molecular weight, as well as certain non-volatile compounds, also exhibit significant oviposition-repellent effects [198]. These effects cannot be fully explained by the odorant-binding type theory. As a result, researchers [196] propose that non-volatile substances, such as azadirachtin and rhodojaponin, induce repellent behavior through a contact type mechanism. This mechanism likely involves the target protein, CSPs, located in chemosensillum of insect’s tarsus or antennae, which mediate oviposition deterrence and behavioral repulsion upon physical contact [199]. Studies on insect CSPs have provided new insights into these mechanisms and hold considerable theoretical and practical significance in repulsion-based pest control. Understanding these molecular interactions could aid in the development of insect behavior regulators that target specific chemical receptors, paving the way for innovative pest control strategies and advancements in pesticide development.

### Molecular markers for insect breeding

Molecular marker-assisted breeding, an emerging technique, applies the theories and tools of molecular biology to enhance conventional breeding practices and screen for traits desirable for both farmers and consumers without involving transgenic approaches [200]. This new technique has been widely employed in the breeding of plant and animal varieties [201]. Insect olfactory binding proteins serve as valuable molecular markers when breeding varieties of economically relevant insect, playing a crucial role in enhancing yield and disease resistance in insects [121, 202]. Studies have shown that Italian bee workers use CSP4 in their antennae to detect larval pheromones such as β-ocimene and alloocimene, which stimulate larval feeding behavior. Based on this discovery, selectively breeding Italian bee strains with high CSP4 expression could enhance royal jelly production [121]. Additionally, research on the selected Italian bees has revealed that individuals exhibiting strong grooming behavior, an important trait for disease resistance, show elevated expression of OBP17 and OBP18 in their antennae. These proteins may help bees recognize pathogenic microorganisms, thereby reducing disease incidence within colonies [202] and ultimately improving honey production. To accelerate the breeding process, researchers have utilized molecular modeling, molecular docking, and phylogenetic analysis to establish rapid screening models for pairing honeybee OBPs with odorant molecules [48, 203]. For example, studies have demonstrated highly stable binding between OBP16 and N-phenyl-2-naphthylamine, 3BJH and crack cocaine, OBP10 and methadone, and OBP24 and ecstasy/marijuana. This research not only provides new insights for selective breeding but also opens new avenues for training bees to detect illicit substances and explosives, enabling practical applications in security and law enforcement [203]. Currently, OBP-based breeding strategies have primarily been applied to economically relevant insects such as honeybees and silkworms [204–206], with no reported cases in other insect species, particularly pests.

### Development of biosensors

Since the discovery of insect olfactory binding proteins, researchers have strived to design efficient biosensors through leveraging their high binding specificity and sensitivity [48, 207]. Recent advances in understanding odor recognition mechanisms in biological systems have accelerated progress in biosensor development [208]. Although the biological materials such as cultured cells [209], olfactory neurons [210], whole tissues [211], ORs [212], and OR peptides [213], are being used as biosensing elements, insect olfactory binding proteins possess distinct advantages that make them the most promising candidates [214, 217, 219, 223]. By expressing insect olfactory binding proteins in model organisms such as bacteria and coupling them to sensing devices using physicochemical methods, these biosensors can detect specific substances with micromolar-level sensitivity [216]. The pioneering utilization of insect olfactory binding proteins in biosensor technology was initially demonstrated in honeybee (*A. mellifera*) OBPs [215], subsequently sparking extensive research has been conducted on the ligand-binding properties of olfactory binding proteins in honeybee. Notably, honeybee OBP2 (ASP2) and OBP14 have been successfully developed into biosensors capable of detecting the changes of odorant molecules in the environment [217, 218]. Similarly, the vinegar fly LUSH demonstrates strong recognition of alcohols, and a biosensor based on this protein has been used to detect Salmonella content in ham and beef [219, 220]. Another biosensor, designed using recombinant AgamOBP1 from the Gambian mosquito, has been applied to detect the characteristic metabolite in *Escherichia coli*, indole, offering a method for analyzing the quality and contamination of drinking water [162]. Moreover, AgamOBP1 mutants exhibit high affinity for substances such as marijuana, ecstasy, and cocaine [221], functioning similarly to honeybee OBPs [203] and presenting promising potential for odor-based biosensing applications. These findings highlight the feasibility and significant practical value of biosensor development based on insect olfactory binding proteins.

Despite these advancements, the widespread application of olfactory biosensors remains limited due to gaps in our understanding of olfactory encoding and the lack of highly effective olfactory sensing elements [48]. One major challenge is the complexity of olfactory encoding, as research on protein ligand-binding spectra remains incomplete. Analysis of known olfactory binding proteins have shown that a single protein molecule can recognize and bind multiple ligands, while a single ligand molecule can also be bound to multiple proteins, resulting in a decrease in detection specificity [53, 54]. Developing biosensors based on these proteins requires identifying highly specific, high-affinity ligand molecules, a task complicated by the structural diversity of ligands. Improving olfactory sensing elements is another key challenge. Even though existing sensor technologies can detect substances at micromolar levels, their sensitivity remains far below that of insect olfactory systems [217, 222]. To address this, researchers have mimicked Gambian mosquito OBP function and developed a biosensor using independent silicon nanowire arrays (SiNWs), achieving detection limits in the parts-per-billion (ppb) range, marking a significant improvement in sensitivity. This approach holds great promise for applications in biomedicine and environmental monitoring [223]. However, further research is needed to enhance detection accuracy and sensitivity to match the capabilities of insect olfaction.

## The unknown

For insect olfactory binding proteins, there is a large amount of information about the binding affinities for a wide diversity of compounds in vitro [2, 54, 80, 95]. But there is a great need for analysis of their functions in vivo and structure–function relationship at the molecular level.

Antennal OBPs are demonstrably capable of binding odorants or pheromones at the molecular level. However, the binding affinities in vitro may not necessarily reflect in vivo due to differing conditions. Although in vitro studies are valuable for predicting OBPs function, these predictions require in vivo validation. Critically, there is little direct evidence that reducing OBPs levels via knockdown/knockout specifically diminishes the electro-physiological response of a sensillum to the same odorants as in vitro [19, 85], making researches involving in vivo test of OBPs function all the more urgent.

Another challenging research direction is to identify ligands in vivo. Certain antennal OBPs are expressed in the absence of olfactory receptor neuron (ORN) dendrites, implying these OBPs (and possibly others) bind ligands other than odorants [19]. Unbiased identification of ligands by isolating OBPs directly from OBP-ligand complex in antennae would uncover unexpected findings in vivo.

Identification of ligands in vivo would inform two critical issues: What do OBPs do after binding ligands, and what happens to the ligands? Both issues may be addressed using physiological analysis conducted in defined sensilla where OBP levels have been experimentally manipulated in a defined way. Ultrastructural analysis of sensilla with manipulated OBP content may also be informative, especially given the exceptionally high concentration of OBPs in the sensilla [92]. Could removal of abundant OBPs affect the ultrastructure of olfactory sensilla? How about removal of a single OBP? Accordingly, a high priority for future research is the construction of “empty sensilla” lacking OBPs using the OBP-to-sensillum map [19]. By using the “empty sensilla”, impacts of total OBPs removal may be investigated across different types of sensilla, with impacts of a single OBP removal also amenable to examination [92].

Current research on insect olfactory binding proteins, though substantial, has yet to fully address key issues due to their structural complexity and dynamic protein-environment interactions. The advent and integration of emerging technologies, such as transcriptomics, RNA interference (RNAi), and CRISPR-mediated gene editing, are profoundly transforming research on these proteins. Transcriptomics enables the highly efficient screening of OBP genes expressed under specific physiological states or ecological contexts, revealing potential functional targets [67, 145]. RNAi allows for the rapid knockdown of OBP expression to preliminarily assess its impact on olfactory behavior [19, 85]. CRISPR technology, meanwhile, facilitates precise gene knockout or knock-in, enabling the direct molecular-level validation of the structure–function relationship of specific OBPs and the investigation of their core role in insect adaptation to diverse odor environments (ecological adaptation). The integration of such approaches would effectively overcome difficulties and challenges inherent in conventional research approaches, significantly advancing our understanding of the complex mechanisms underlying the functions of insect olfactory binding proteins in insect olfaction.

## Summary and outlook

Insect olfactory system is remarkably sensitive, largely due to the role of olfactory binding proteins. To date, a large number of insect olfactory binding proteins have been identified, exhibiting structural complexity, functional diversity, and widespread distribution across nearly all insect sensilla [196]. In-depth studies of these proteins would provide valuable insights into how insects interact with environmental chemical cues, offering a deeper understanding of the molecular mechanisms underlying their signal perception and behavioral responses. Such researches hold considerable practical value in fields such as pest management, insect breeding, and biosensor development. Despite these advances, many aspects of insect olfactory binding proteins, including structural properties, physiological functions, information-transfer mechanisms, and signal transduction, remain insufficiently explored. Future research should prioritize elucidating the structure–function relationships of these proteins, their roles in olfactory signaling pathways, mechanisms of action in the olfactory system and the role of OBPs genetic variation in olfactory sensitivity and behavior in different species. The continued advancement of molecular biology and chemical analysis techniques, coupled with the integration of large-scale models and artificial intelligence, is expected to expand the scope of traditional biological research. These developments will not only enhance our understanding of insect olfaction but also open new avenues for innovative applications in both fundamental and applied sciences, such as the construction of cross-species OBP large-scale model.

## Data Availability

Not applicable.

## Abbreviations

OBP: Odorant-binding protein
CSP: Chemosensory protein
NPC2: Niemann-Pick type C2 protein
OR: Odorant receptor
ODE: Odorant-degrading enzyme
PN: Projection neuron
MB: Mushroom body
LH: Lateral horn
GOBP: General odorant-binding protein
PBP: Pheromone-binding protein
ASP: Antennae-specific protein
NMR: Nuclear magnetic resonance
PDB: Protein data bank
pH: Potential of hydrogen
PDE: Pheromone-degrading enzyme
RNAi: RNA interference
PEA: Phenylethyl alcohol
LPS: Lipopolysaccharide
PSM: Plant secondary metabolite
SiNW: Silicon nanowire array
ppb: Parts-per-billion
